# A challenging high-risk surgery for necrotizing pneumonia in a right bilobed lung

**DOI:** 10.1186/s12887-023-03999-y

**Published:** 2023-04-13

**Authors:** Turyalai Hakimi, Mohmand Mangal, Mohammad Akbar Ibrahimi, Mansoor Aslamzai, Khesrow Ekram, Mohammad Hussain Shiwa, Zamaryalai Hakimi, Abdul Tawab Noory, Abdul Ghafar Hamdard, Sultan Ahmad Halimi, Mohammad Anwar Jawed

**Affiliations:** 1 Department of Pediatric Surgery, Kabul University of medical science, Maiwand teaching hospital, Kabul, Afghanistan; 2 Department of Pediatrics, Kabul University of medical science, Maiwand teaching hospital, Kabul, Afghanistan; 3 Department of Neonatology, Kabul University of medical science, Maiwand teaching hospital, Kabul, Afghanistan; 4 Department of General Surgery, Kabul University of medical science, Ali Abad teaching hospital, Kabul, Afghanistan; 5 Department of Infectious disease, Kabul University of medical science, Ali Abad teaching hospital, Kabul, Afghanistan; 6 Department of Dermatology, Kabul University of medical science, Maiwand teaching hospital, Kabul, Afghanistan; 7 Department of Pathology, Kabul University of medical science, Kabul, Afghanistan

**Keywords:** Lung necrosis, Antibacterial therapy, Lung consolidation, Respiratory distress, Wedge resection

## Abstract

**Background:**

Necrotizing pneumonia is rare in children and is one of the most serious complications of a lung infection caused by antibiotic failure. We present a 12-year-old leukopenic child with a long-lasting lung infection, presenting as having a lung hydatid cyst, but diagnosing with necrotizing pneumonia in the right bilobed lung. Failure to medical treatment and ongoing leukopenia justified surgical intervention with positive results.

**Case presentation:**

The patient was referred to our teaching hospital’s pediatric surgery department. He had previously been diagnosed with intestinal tuberculosis (TB) and received anti-TB treatment. On referral to our hospital, the patient was suffering from restlessness, frequent coughing, fever, vomiting, and diarrhea. Following the completion of the clinical work-up, a blood test revealed leukopenia (white blood cell count of 2100/microliter), a normal platelet count, and a lesion in the right lung. Computerized tomography scanning (CT-Scan) image reported a lung hydatid cyst. In the pediatrics ward, a broad-spectrum antibiotics regimen with triple-antibiotic therapy (linezolid, vancomycin, and metronidazole) was instituted and continued for a week with no response, but worsening of the condition. In the pediatric surgery ward, our decision for surgical intervention was due to the failure of medical treatment because of a pulmonary lesion. Our team performed right lung upper lobe anterior segment wedge resection due to necrotizing pneumonia and followed the patient 45 days post-operation with a reasonable result.

**Conclusion:**

Living in remote rural areas with low resources and inaccessibility to proper and specialized diagnostic and treatment centers will all contribute to an improper diagnosis and treatment of lung infection. In total, all of these will increase the morbidity and mortality due to lung necrosis in the pediatric population, regardless of their age. In low-resource facilities, high-risk patients can benefit from surgical intervention to control the ongoing infection process.

## Introduction

Pneumonia is a clinical condition where the lower respiratory tract epithelium below the larynx is invaded through inhalation, or hematogenous spread [1]. The annual estimation of pneumonia reaches 120 million cases worldwide, with the consequent 1.3-million deaths [2]. In developing nations, children under 2-years of age account for nearly 80% of pneumonia related deaths [3]. Necrotizing pneumonia is one of the severe complications of pneumonia and is characterized by continuing pneumonic illness in a previously healthy child despite proper antibiotic therapy [4]. Diagnosis is made by imaging and pathological analysis. Chest radiographs show pulmonary consolidation with one or more small, thin-walled cavities, whereas pathologic examination shows pulmonary inflammation, alveolar consolidation, and thrombosis of intrapulmonary vessels, and consequent necrosis with multiple small cavities. Reduced blood flow due to thrombotic vessels is thought to be the cause of pulmonary tissue destruction [4, 5].

## Case presentation

A 12-year-old cachectic child was referred from a south-border province of Kandahar to our pediatric service complaining of fever, restlessness, shortness of breath, anorexia, and diarrhea. The patient was born to a consanguineous couple with unremarkable antenatal history. The patient developed diarrhea and fever at the age of one year, according to his uncle explanation (as attendant). Since then, with the frequent advice of local physicians, no remedy was achieved, but the condition remained unchanged. During this time, food intolerance and undigested food passage in the stool were also noted by his family. His uncle, added that he took the child two times outside the country for better treatment at the ages of 4 and 9 years respectively. The patient also received anti-TB treatment at the age of 11-year. The reason for his referral to the capital, and then to our pediatric service, was ongoing deterioration of his condition.

**Fig. 1:**
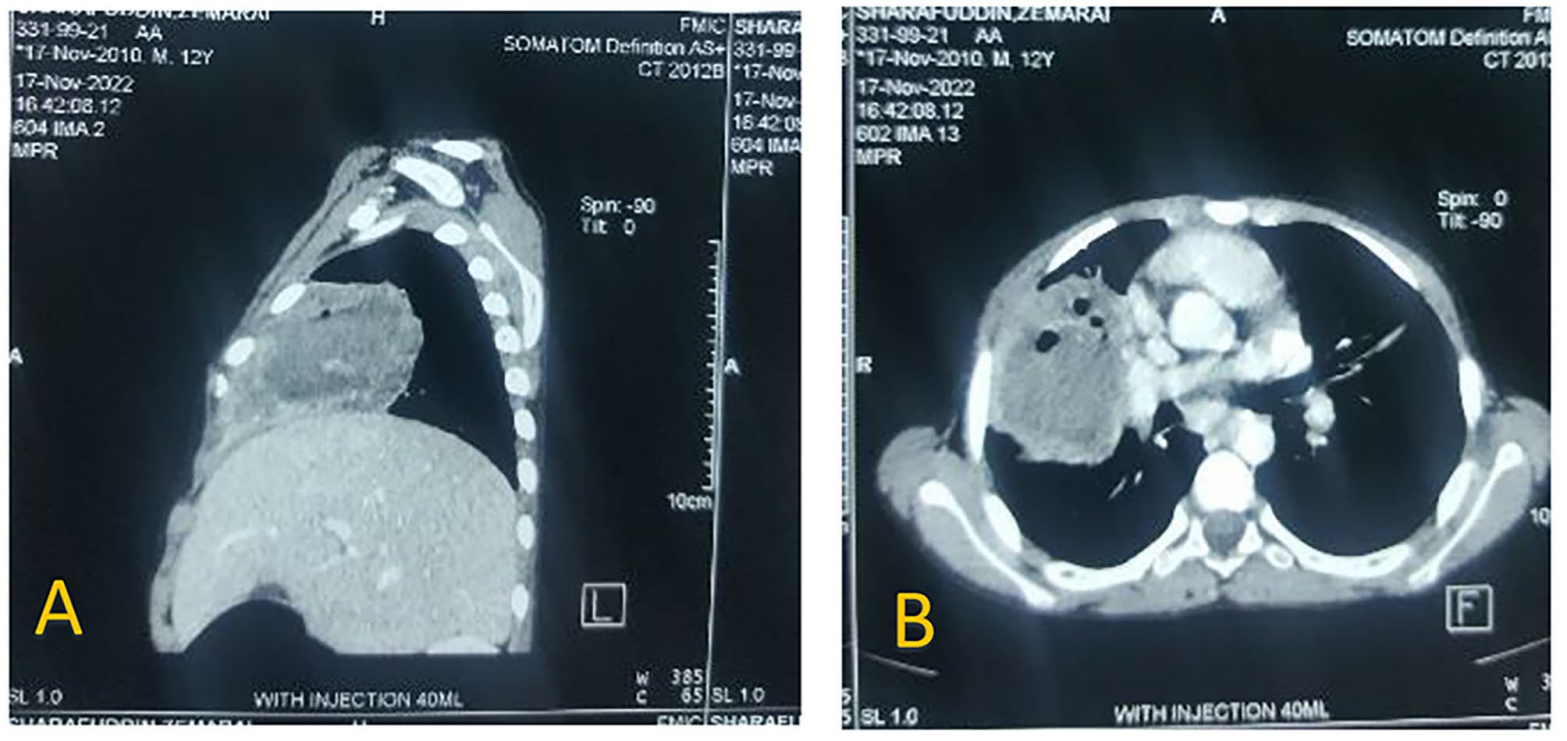
**A**) Lateral view and **B**) Cross-sectional view of the necrotizing pneumonia

On physical exam, the patient looked cachectic, with keratoderma on his both palmoplantar surfaces. With auscultation, respiratory sounds decreased on the right side. In reference to the above-mentioned history, the patient was admitted to the pediatrics ward on the suspicion of TB superimposed by a chest infection, with him being put on triple-antibiotic (linezolid, vancomycin, and metronidazole) therapy until the diagnostic report of TB. Appropriate tests for TB were all negative, but WBC was recorded at 2100/microliter and a chest X-ray (CXR) revealed a right lung middle part density. A chest CT scan was ordered to confirm the diagnosis, which reported a right lung hydatid cyst (Fig. 1A and B). Here, since the leukopenia was worsening with no evident response, the patient was referred to our pediatric surgery ward on the suspicion of ongoing leukopenia, which may be aggravated by an existing lesion, and admitted.

**Fig. 2:**
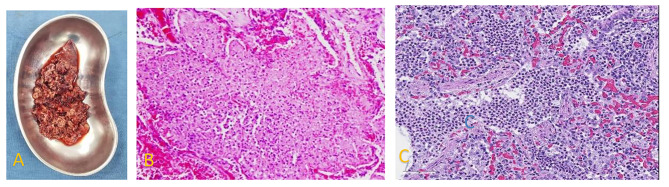
**A**) resected specimen for pathologic analysis, **B** and **C**) acute necrotizing pneumonia: micrographs revealed dense, purulent exudate with septal wall necrosis in both figures

Our team discussed the patient and reordered the complete blood count (CBC), which revealed ongoing leukopenia (WBC decreased to 1200/microliter) but a normal platelet count. The justification for surgical intervention was a lung lesion that may be the cause of medical treatment failure. The patient was scheduled for surgery and was put on non-peroral the night before surgery. Our approach was through the right anterolateral thoracotomy incision. Using a chest spreader, we exposed the right lung and noted a bilobed right lung (absent middle lobe) with a discolored area in the lower part of the anterior segment. With careful tissue handling, our team performed anterior segment wedge resection using non-absorbable suture. A chest drain was left inside, and the patient tolerated the operation. The resected specimen (Fig. 2A) was sent to our university’s pathology department, which diagnosed and confirmed necrotizing pneumonia (Fig. 2B, and C). Following 8 post-operative days, the patient was discharged. After 45 days of follow-up, the patient was rechecked with a considerable positive result: a normal CBC result and appetite improvement with weight gain. 

## Discussion and conclusions

The first case of necrotizing pneumonia (NP) in children was reported in 1994, with a gradual increase until now. The most common pathogens implicated in pediatrics with NP are pneumococci and Staphylococcus aureus. NP is an intermediate condition between pulmonary abscess and pulmonary gangrene, where the latter is mostly accompanied by empyema and bronchopleural fistulae (BPF) [5]. In 0.8–7% of cases, community-acquired pneumonia (CAP) may complicate NP [6]. The incidence of complicated pneumonia has risen in the last two decades; however, the condition is still uncommon and occurs in less than 1% of pediatric patients with CAP [7]. Most of the children are under 5 years old, and the clinical manifestations of NP include those of pneumonia, such as fever, cough, tachycardia, chest pain, and localized chest signs, which show dullness by percussion with decreased breath and/or bronchial breathing sounds. Symptoms have long existed before presentation, and the patient looks overly sick with persistent fever, respiratory distress, and non-responding clinical and radiographic signs of ongoing pneumonia [8]. Empyema coexists in 60–100% of cases, and BPF is diagnosed on a chest radiograph. Continuing (> 24 h) gas leakage from chest tubes is considered a common (17–67%) complication [9].

Children with pneumonia who remain unwell for 72 h despite appropriate antimicrobial therapy should be suspected for NP, especially if the BPF or loculated empyema is present despite drainage and surgical management without improvement [10, 11]. Extra-pulmonary site infections, such as those of the skin, soft tissue, and musculoskeletal system, should also be sought. Sometimes, following presentation, the condition of children with NP deteriorates to severe sepsis, presenting as septic shock, multi-organ failure, and hypoxic respiratory failure [17]. Reduced peripheral white blood cell (WBC) counts are concerning and have been linked to S. aureus infections in the past [12]. CXR will show underlying pneumonia with coexistent parapneumonic effusion (PPE) and shifted mediastinum, and if one or more small lucencies or pneumatoceles are seen in the 4–8-day interval of hospitalization, this imaging modality is diagnostic in 27–41% of pediatric cases of NP. In the late stage of NP, the lesion became more visible when the necrotic fluid drained into the communicating bronchi and was replaced by gas [13].

Contrast-enhanced chest CT-scan evaluates lung parenchymal changes that are not visible on CXR and detects underlying congenital lung malformations; therefore, this modality is superior to CXR in the diagnosis of NP [14, 15]. The diagnostic highlights are weak or absent vascularity, loss of pulmonary architecture, and cavity formation. Multiple small gas or fluid-filled, thin-walled cavities are usually seen involving only a single lobe. As the lung continued to liquefy, the multiple small cavities may coalesce and form larger cavities, including gas-filled pneumatoceles, which develop in the later stages of NP [16, 17].

A number of disorders will cause pulmonary cavities, but infection-induced lung cavities and infected congenital lung defects along with traumatic pseudocysts should always be considered in the differential diagnosis of children with NP [18]. Differentiating NP from lung abscess is clinically important since the underlying causes and management can differ. A simple lung abscess has an inactive course of fever and cough that can last for weeks. Investigational delay may lead to complications, which are represented by multiple thin-walled cavities in the NP that may fuse to form one or more contrast-enhancing, thick-walled abscesses containing gaseous fluid levels within the pulmonary consolidated regions [19].

Management of NP includes administration of supplemental oxygen to relieve hypoxia, analgesics for reducing pleuritic pain and consequent improvement of ventilation, prolonged antibiotic therapy, and reduction of mass effects or intrathoracic pressure by draining gas and/or intrapleural fluid [14, 15].

In a healthy and fully immunized child, the treatment option is antibiotics to cover gram-positive organisms, particularly pneumococci, S. aureus, and S. pyogenes, as with empyema. The first-line recommended treatment for children with pneumonia includes intravenous (IV) penicillin or ampicillin and is switched to beta-lactam anti-staphylococcal antibiotics, such as oxacillin or flucloxacillin, in those who are hospitalized due to severe but uncomplicated community-acquired pneumonia (CAP) [20, 21]. In cases of suspicion of methicillin-resistant Staphylococcus aureus (MRSA), confirmed by culture, and conditions (e.g., local prevalence > 10%, ethnicity, recent personal or household history of skin infections), appropriate antibiotics should be used. Although, vancomycin penetration in the pulmonary parenchyma is poor, it has a 20% treatment failure rate in the case of MRSA pneumonia if given as monotherapy; hence, until MRSA is confirmed, a beta-lactam anti-staphylococcal antibiotic should be part of the management protocol [22]. However, the addition of linezolid, clindamycin, or rifampicin, which are capable of inhibiting protein synthesis (including toxin production), in cases of infection with *S.* aureus or S. pyogenes infections, has superior results, but high-level evidence for this regimen is lacking [21]. In cases of suspicion for mycoplasma pneumonia (MP), macrolides such as IV clarithromycin or azithromycin are added but do not replace the antibiotics that are active against pneumococci and S. aureus [23]. More often, in children with NP, the microbiology result is negative, and the resistance of respiratory pathogens against macrolide is high [24]. Finally, if the child is unimmunized against H. influenzae type b (Hib), immunocompromised, or the infection is suspected to be hospital-acquired, the best initial empiric antibiotic therapy includes extensive gram-negative coverage with the addition of a third or fourth generation cephalosporin. The desired course of treatment with antibiotics in NP ranges from 13 to 42 days, with an average duration of 21 days. Following the normalization of the body temperature, inflammatory markers, feeding tolerance, and respiratory distress, the IV antibiotics should be switched to oral antibiotics and followed for 10–14 consecutive days [20].

Surgical intervention is rarely recommended to avoid the risk of bronchopleural fistula (BPF). Although loculated empyema can cause mass effects and lead to hemodynamic and ventilation instability, necessitating surgery. A large pyopneumothorax and tension pneumatocele will all necessitate surgery. Furthermore, necrosis of the lung is only detected during surgery. In cases with large parapneumonic effusion (PPE) and pyopneumothorax, chest tube drainage is enough, but some experts recommend the instillation of intrapleural fibrinolysis in cases of loculated empyema. However, chest tube drainage > 7 days and fibrinolysis may increase the risk of BPF and failure in 30% of empyema patients [20]. If fever, signs of sepsis, and/or respiratory distress persist in spite of chest tube insertion with or without fibrinolytic therapy and frequent imaging shows ongoing intrapleural collections, surgical intervention such as video-assisted thoracoscopic surgery or mini-thoracotomy for the debridement of pyogenic material around the lung (decortication), breakdown of loculations, and removal of pus may be indicated. If the symptoms due to the underlying NP are mild, continuing IV antibiotics without surgery is ideal. When progressive lung necrosis is the issue, surgical intervention in the form of segmental or lobar resection or pneumonectomy is the treatment of choice, which is rarely required in children. Poor penetration of antibiotics into pulmonary hypoperfused regions and into cavitating lesions leads to delayed bacterial clearance, tissue necrosis, and ongoing inflammation; therefore, children with NP will have intermittent fever for several days despite proper antimicrobial therapy and chest drainage [25]. Some authors recommend reviewing the child in 2 weeks following discharge from the hospital and then in 6–8 weeks and 6 months at minimum.

Prevention of NP depends on the CAP and its severity [26]. Decreases in CAP are related to a combination of improvements in housing, water supply, and hygiene; preferable indoor air quality; decreased parental tobacco smoking; increased education; breastfeeding rates and nutrition; and extensive healthcare access along with vaccine uptake [27]. To reach this goal, pertussis, measles, Hib, and pneumococcal conjugate vaccines should be received [28].

The mentioned case involved an older patient, contrary to the literature (under 5-year-old children), with a long-lasting chest infection. The patient was treated for the suspicion of a chest infection and TB, which were treated in a local clinic and neighboring country. The patient remained unresponsive with a long-lasting disease and was referred to our teaching hospital’s pediatric surgery department. Even in a reputable medical facility in the capital, an advanced imaging modality (chest CT-Scan) was misreported. The justification for surgical intervention was medical treatment failure and existing lung lesion. The reason for the diagnostic delay seems to be due to a lack of facilities, such as the absence of equipped medical centers. Socioeconomic factors are also implicated and cause the patient to rely on primary care on a local basis. In low-resource settings, however, a lack of professionals and arbitrary treatment will mask the definite diagnosis, and broad-spectrum antibiotics (single, double, or triple combination) may all play a role in weakening the patient defense system and increasing resistance to the adopted international treatment protocol.

With the institution of the above-mentioned triple antibiotics, the WBC count decreased from 2100 to 1100/microliter, which may be due to linezolid, but for the prevention of hospital-acquired infection, we had to rely on this protocol. *(Infection prevention is our hospital priority, but in most of the developing nations, the international standards are not met accordingly.)* During the post-operative course of the hospital stay, the decreased WBC level was managed by the transfusion of 20ml of iso-group fresh blood per kg every other day in three sessions, which resulted in a considerable boost of WBC. Our patient also exhibited extrapulmonary involvement of the skin (keratoderma of palmoplantar surfaces), which could be due to a weak defense system and was treated accordingly. On the 45th post-operative day of monitoring, all the blood markers returned to normal, but the patient complained of mild signs and symptoms of a common cold (due to the winter cold weather); therefore, he was advised on appropriate medication and rehabilitation. From the first day of admission, our patient’s WBC level ranged between 2100, 1800, 1200, and 2600 µl, making him a high-risk patient for surgery. Additionally, hospital-acquired pneumonia (HAP) and low-quality antibiotics will all play their roles.

## Data Availability

The datasets used in the current article, are available from the corresponding author on reasonable request.

## Abbreviations

NP: Necrotizing pneumonia
CXR: Chest X-ray
TB: Tuberculosis
CT: Computerized tomography
IV: Intravenous
Hib: Haemophilus influenzae type b
BPF: Bronchopulmonary fistula
CAP: Community acquired pneumonia
PPE: Parapneumonic effusion
MRSA: Methicillin-resistant Staphylococcus aureus
MP: Mycoplasma pneumonia
HAP: Hospital acquired pneumonia
